# DOMAIN-SPECIFIC AGE STEREOTYPES AND ADULTS’ PERCEIVED FUTURE TIME EXPERIENCES

**DOI:** 10.1093/geroni/igz038.2327

**Published:** 2019-11-08

**Authors:** Han-Jung Ko, Yen-Pi Cheng

**Affiliations:** 1 Central Michigan University, Mount Pleasant, Michigan, United States; 2 Independent Scholar, San Jose, California, United States

## Abstract

Experiencing ageism has been shown to affect older adults’ outlook for the future (Barber & Tan, 2018). However, ageism is a multi-faceted construct in addition to positive and negative age stereotypes. In this study, we examined to what extent domain-specific age stereotypes are related to different aspects of a person’s perceived future time experiences. A total of 646 participants (aged 18 to 83) were recruited from a U.S. mid-Western public university for an online anonymous survey. Age stereotypes were assessed in eight life domains of family, friends, religion, leisure, lifestyle, finance, work, and health (Kornadt & Rothermund, 2011). Future time experiences were assessed in four aspects, including perceived openness and concreteness of future, acceptance of life’s finitude, preoccupation with the past, and experiences of obsolescence (Brandtstädter & Wentura, 1994). Age stereotypes in the family domain were associated with one’s perceived openness and concreteness of future and preoccupation with the past. The religion and financial domains were both associated with perceived openness and concreteness of future and acceptance of life’s finitude. Moreover, the older the participants were, the more they accepted life’s finitude. Surprisingly, none of the domains significantly predicted one’s perceived future experiences of obsolescence. Unlike the common finding that people hold negative age stereotypes due to physical and mental declines, age stereotypes in the health domain did not predict any aspects of the future time experiences. Our findings highlight the importance of distinguishing ageism across domains in order to improve one’s perceived future time experiences.

